# GOALS OF CARE IN A MEDICAID HCBS PROGRAM: VARIATIONS BY RACE, ETHNICITY, AND DEMENTIA

**DOI:** 10.1093/geroni/igae098.3532

**Published:** 2024-12-31

**Authors:** Julie Robison, Victor Elisha, Kathy Kellett, Kristin Baker, Richard Fortinsky

**Affiliations:** University of Connecticut, Farmington, Connecticut, United States; University of Connecticut, Farmington, Connecticut, United States; UConn Health, Farmington, Connecticut, United States; UConn Health, Farmington, Connecticut, United States; University of Connecticut, Farmington, Connecticut, United States

## Abstract

Medicaid-funded home and community-based services (HCBS) for older adults aim to prolong independent living despite physical and cognitive disabilities. HCBS person-centered care plans ideally align services provided with recipients’ goals of care. Medicaid-eligible HCBS populations are racially and ethnically heterogenous, with multiple chronic conditions. This study aims to determine how living with dementia, and racial and ethnic group membership, are associated with meeting self-identified goals of care and person-centered outcomes based on their HCBS-related experiences. 148 ethnically diverse adults ages 65+ with and without dementia were interviewed 9-10 months after enrolling in Connecticut’s Medicaid HCBS waiver program about the goals they set at enrollment. Structured interviews were conducted in person or virtually, based on respondent preference. Assistant or proxy unpaid caregiver respondents participated when warranted. 90% of respondents said they had as much input as they wanted in setting their goals. 76% felt they had met their goal, including 49% doing better than they had expected, and 16% reporting changing their goal. 83% said that thinking about goals was helpful in planning future care and many had discussed their goals with another health care provider (45%) or friends and family (76%). Goal achievement was equally likely for people with dementia compared to people without dementia as well as between race and ethnicity groups. However other aspects differed both by dementia status and race/ethnicity. Examples of specific goals for various subgroups will be presented.

